# A comprehensive review of acute cardio-renal syndrome: need for novel biomarkers

**DOI:** 10.3389/fphar.2023.1152055

**Published:** 2023-05-23

**Authors:** Abhi Dutta, Shubham Saha, Ajay Bahl, Anupam Mittal, Trayambak Basak

**Affiliations:** ^1^ School of Biosciences and Bioengineering, Indian Institute of Technology (IIT)-Mandi, Mandi, Himachal Pradesh, India; ^2^ BioX Center, Indian Institute of Technology (IIT)-Mandi, Mandi, Himachal Pradesh, India; ^3^ Department of Cardiology, Postgraduate Institute of Medical Education and Research, Chandigarh, India; ^4^ Department of Translational and Regenerative Medicine, Postgraduate Institute of Medical Education and Research, Chandigarh, India

**Keywords:** cardiorenal syndrome, heart failure, fibrosis, proteomics, biomarkers, kidney failure

## Abstract

Cardiorenal syndrome represents a wide-spectrum disorder involving the heart and kidneys as the primary affected organs. India has an increasingly high burden of acute CRS, coinciding with the rise in global statistics. Up to 2022, approximately 46.1% of all cardiorenal patients have been diagnosed with acute CRS in India. Acute CRS involves a sudden deterioration of kidney functionalities, referred to as acute kidney injury (AKI) in acute heart failure patients. The pathophysiology of CRS involves hyperactivation of the sympathetic nervous system (SNS) and the renin-angiotensin-aldosterone system (RAAS) following acute myocardial stress. The pathological phenotype of acute CRS is associated with perturbed inflammatory, cellular, and neurohormonal markers in circulation. These complications increase the risk of mortality in clinically diagnosed acute CRS patients, making it a worldwide healthcare burden. Hence, effective diagnosis and early prevention are crucial to prevent the progression of CRS in AHF patients. Present biomarkers, such as serum creatinine (sCr), cystatin C (CysC), glomerular filtration rate (GFR), blood urea nitrogen (BUN), serum and/or urine neutrophil gelatinase-associated lipocalin (NGAL), B-type natriuretic peptide (BNP), and NT-proBNP, are clinically used to diagnose AKI stages in CRS patients but are limitedly sensitive to the early detection of the pathology. Therefore, the need for protein biomarkers is emerging for early intervention in CRS progression. Here, we summarized the cardio-renal nexus in acute CRS, with an emphasis on the present clinicopathological biomarkers and their limitations. The objective of this review is to highlight the need for novel proteomic biomarkers that will curb the burgeoning concern and direct future research trials.

## 1 Introduction

Inter-organ cross-talks are fundamental to the healthy physiological functioning of the body, wherein pathological injury to one organ can cause the acute or chronic dysfunction of another. The heart and kidney reciprocally influence one another through diverse hemodynamic and non-hemodynamic pathways necessary for cardiovascular homeostasis. A maladaptive physiological nexus between the heart and kidneys has recently been characterized as cardio-renal syndrome (CRS) (Costanzo, 2020). The bidirectional overlap of cardiac and kidney disorders caused by one damaged primary organ is broadly described to be multifactorial (Ronco et al., 2008). The National Heart, Lung, and Blood Institute attempted the first definition of the umbrella term CRS in 2004: “the *result of interactions between the kidneys and other circulatory compartments that increase circulating volume, exacerbating symptoms of heart failure (HF) and disease progression*” (Ronco et al., 2010). However, following this, in the 2008 Acute Dialysis Quality Initiative (ADQI) consensus, Ronco et al. broadly classified CRS into five subtypes mostly based on the primary organ dysfunctioning and the acute or chronic nature of events (Ronco et al., 2008). Type 1 or acute CRS is the rapid progression of acute kidney injury (AKI) or dysfunction in the setting of acute heart failure (AHF). Type 2 or chronic CRS is chronic heart failure underlining the development of chronic kidney disease (CKD). CRS types 3 and 4 are considered reno-cardiac syndromes, caused by acute and chronic kidney dysfunction underlying adverse cardiovascular pathologies such AHF and chronic heart failure (CHF), respectively. In type 5 CRS, systemic diseases such as amyloidosis, sepsis, and cirrhosis cause failing heart and kidney functionalities (Rangaswami et al., 2019). The reported incidence of CRS varies significantly among different subtypes and also among different age groups. AKI is the most prevalent and occurs in a large fraction of clinical acute decompensated heart failure (ADHF) or heart failure (HF) patients (Bagshaw et al., 2010; Hoste et al., 2018). Kidney dysfunctionality in AHF patients is associated with higher mortality and morbidity. AKI is defined and staged by three different concurrent groups, i.e., RIFLE (risk, injury, failure, loss of kidney function, and end-stage kidney disease), AKIN (acute kidney injury network), and KDIGO (kidney disease: improving global outcomes) are widely used (Roy et al., 2013). These variable diagnosis criteria for AKI limit the early diagnosis of CRS. However, despite variable diagnosis patterns, patients hospitalized with ADHF or acute coronary syndrome (ACS) and with varied comorbid conditions showed 20%–75% and 3%–43% incidences, respectively, of developing CRS type 1, accelerating the morbidity (Bagshaw et al., 2010; Fernando et al., 2021). AKI is also one of the strongest risk factors among cardiac surgery patients, with a prevalence of 22.3% (95% CI 19.8–25.1), and is associated with adverse outcomes (Hoste et al., 2018). Increased mortality in cardiac surgery patients is further associated with renal dysfunctionalities requiring dialysis (Mishra et al., 2005). In a recent study, ∼25% of all patients admitted with ADHF developed kidney dysfunctionalities that occurred secondary to the cardiac onset (Hoste et al., 2018). Interestingly, acute CRS is prevalent (∼16%) in patients admitted with AKI (Seckinger et al., 2022).

Acute CRS is the most prevalent form worldwide with an incidence rate of 27%–50% among all CRS types, albeit supported by a limited number of clinical studies (Gigante et al., 2014; Hu et al., 2016; Uduman, 2018; Prothasis et al., 2020), and is associated with increased detrimental outcomes in CRS patients (Gigante et al., 2014). In a prospective cohort study in India, children hospitalized with CRS showed a 40.3% incidence of the acute form of CRS, the highest among all types (Athwani et al., 2017). CRS (both acute and chronic) accounts for the highest mortality among patients admitted with either HF or CKD or CRS (Halimi et al., 2022), thus demanding a proper early diagnosis. Moreover, clinical reports suggest increased mortality in patients admitted with acute CRS compared to that of patients with only cardiovascular dysfunctionalities such as AHF or ADHF, indicating progression time as a risk factor (Ronco et al., 2010; Shirakabe et al., 2013). Another critical factor in acute CRS patients is the onset timing of AKI. Early onset is associated with a higher rate of hospitalisation, whereas the proportion of hospitalized patients with late onset AKI is significantly lower (Di Lullo et al., 2017). Recent studies reported that in-hospital mortality of early AKI patients, i.e., patients having or developing AKI within 5 days of admission, is 13.8% compared to 11.8% in those who developed AKI at a later stage (Shirakabe et al., 2013).

To date, multiple studies have reported the prevalence, risk factors, and outcomes of CRS. However, an increasing trend of acute CRS as the common cause of adverse outcomes and mortality among ADHF and ACS patients has been reported worldwide (Bagshaw et al., 2010; Fernando et al., 2021). Particularly, there is an alarming rise in the diagnosis of acute CRS in India. Hence, it is critical to understand not only the prevalence or overall burden but also the evaluation of clinical outcomes and diagnostic biomarkers to recognize important research gaps for on-time diagnosis and therapeutics of acute CRS patients. In this review, we summarized the prevalence, clinical outcomes, association, and prospects of protein biomarkers for predicting acute CRS with special reference to the Indian population.

## 2 Prevalence of acute CRS (type 1)

The common onset of acute CRS involves acute decompensated heart failure (ADHF), cardiac surgery (CS), and acute coronary syndrome (ACS) associated with impaired renal function. Patients admitted with ADHF developing either AKI or worsening renal function (WRF) have a five times higher mortality risk (Vandenberghe et al., 2016). Despite differences in defining AKI and population heterogeneity, ∼25%–33% of ADHF patients develop acute CRS (Roy et al., 2013).

**FIGURE 1:**
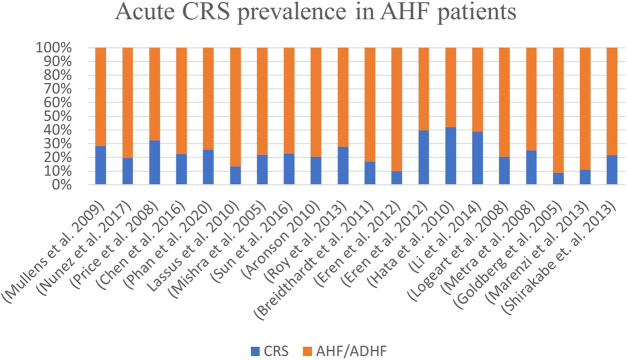
CRS prevalence in India. Proportion of acute CRS in the Indian population.

Worldwide, the prevalence of acute CRS in patients admitted with acute heart failure ranges from 10% to 71% across studies, with an average of ∼32% (Figure 1).

**FIGURE 2:**
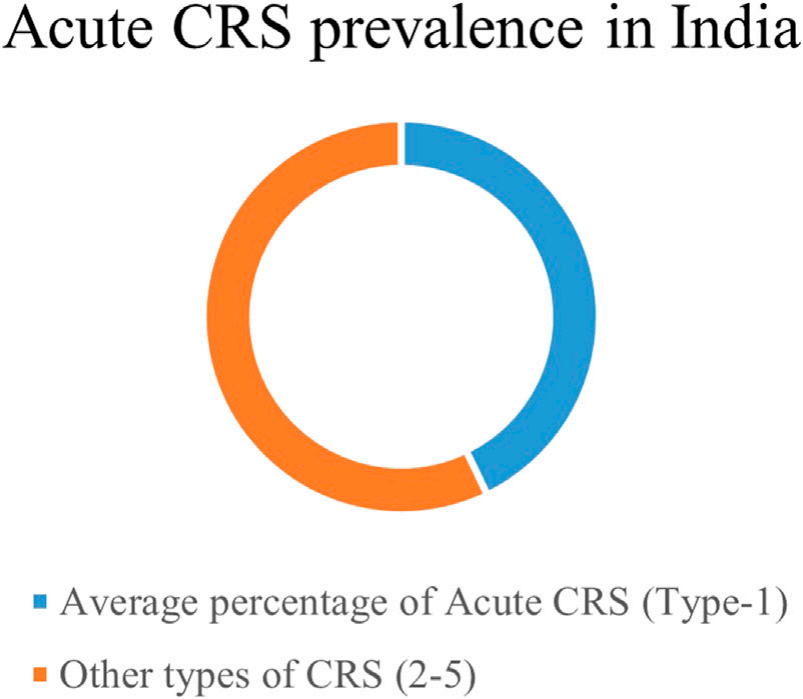
Burden of CRS in symptomatic patients with acute heart failure worldwide. A consensus study from 2005 to 2020.

**FIGURE 3:**
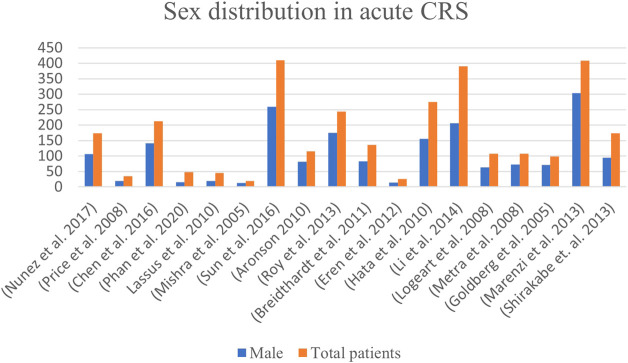
Gender-based distribution of CRS patients in acute heart failure patients worldwide.

In India, a single-centred cross-sectional study evaluating the prevalence of CRS subtypes among CRS patients reported acute CRS to be the most prevalent among CRS patients (Prothasis et al., 2020). Across studies, 41.6% of CRS patients are acute (type 1) in nature among all forms of CRS (Figure 2; Table 1). Of note, hypertension, diabetes mellitus (DM), myocarditis, and CKD are the most common and prevalent risk factors in acute CRS patients in India (Tandon et al., 2013; Prothasis et al., 2020; Reddy et al., 2020) (Figure 3).

**TABLE 1:** Prevalence of acute CRS among cardio-renal or reno-cardiac patients in India.

Total CRS patients	Acute CRS or type 1 (n), (%)	Common risk factor	References
96	47, 48.96%	Hypertension (47.9%)	Prothasis et al. (2020)
50	23, 46%		Shah et al. (2016)
67 (Children)	27, 40.3%	Myocarditis (40.7%)	Athwani et al. (2017)
460	156, 34%	CKD, diabetes mellitus	Fernando et al. (2021)
48	28, 58.34%	Hypertension, diabetes mellitus, cardiogenic shock	Tandon et al. (2013)
158	85, 53.8%	DM (43.5%), hypertension (76.5%)	Reddy et al. (2020)

## 3 Pathophysiology of acute CRS

According to a 2008 classification, Ronco et al. (2008) defined CRS type 1 or acute CRS as rapid worsening of cardiac function leading to kidney injury (Ronco et al., 2008). The pathophysiology of CRS is a multifaceted process and is diagnosed generally at late stages. It causes higher mortality compared to heart failure patients without AKI (Damman et al., 2007). Although precise mechanistic insights are lacking, a widely accepted conceptual framework for the development of acute CRS involves hemodynamic and nonhemodynamic connections (Virzi et al., 2014).

### 3.1 Hemodynamic coupling of heart and kidney

Hemodynamic responses involving multiorgan crosstalk initiated in either the heart or kidney involve activation of the classical renin-angiotensin-aldosterone system (RAAS) and the sympathetic nervous system (SNS) (Braam et al., 2014). The pathogenesis of CRS is multifaceted and involves the activation of hemodynamic signalling, SNS activation, and inflammatory and immune responses, with loss of redox homeostasis (Di Lullo et al., 2017; Rangaswami et al., 2019). Activation of one or another synergistically activates others in a vicious cycle (Bongartz et al., 2004).

The pathophysiological onset of acute CRS is caused by reduced cardiac output (CO) and arterial underfilling (Grodin et al., 2016). In patients with ADHF, static increases in volume overload lead to elevated central venous pressure (CVP). Elevated CVP is directly related to renal dysfunctionalities (Prastaro et al., 2022). Increased CVP leads to depressed renal venous pressure (RVP), creating a low flow state that reduces the blood flow gradient to kidneys and leading to renal hypoperfusion (Bock and Gottlieb, 2010; Prastaro et al., 2022). In patients with reduced cardiac functionalities, the low-flow state occurs due to kidney artery under-filling, resulting in a reduced eGFR (glomerular filtration rate) and reduced renal functionalities, which are associated with higher mortality (Damman et al., 2009; Fu et al., 2021). Reduced renal functionalities account for acute kidney injury (AKI), worsening renal function (WRF), and ischemic injury of glomerular and renal tubules, consequently worsening cardio-renal syndrome. A reduction in GFR leads to increased serum creatinine (sCr) compared to the baseline (≥25%), and is used as a biomarker for all type of CRS.

#### 3.1.1 Renin-Angiotensin-Aldosterone System (RAAS) pathways

**FIGURE 4:**
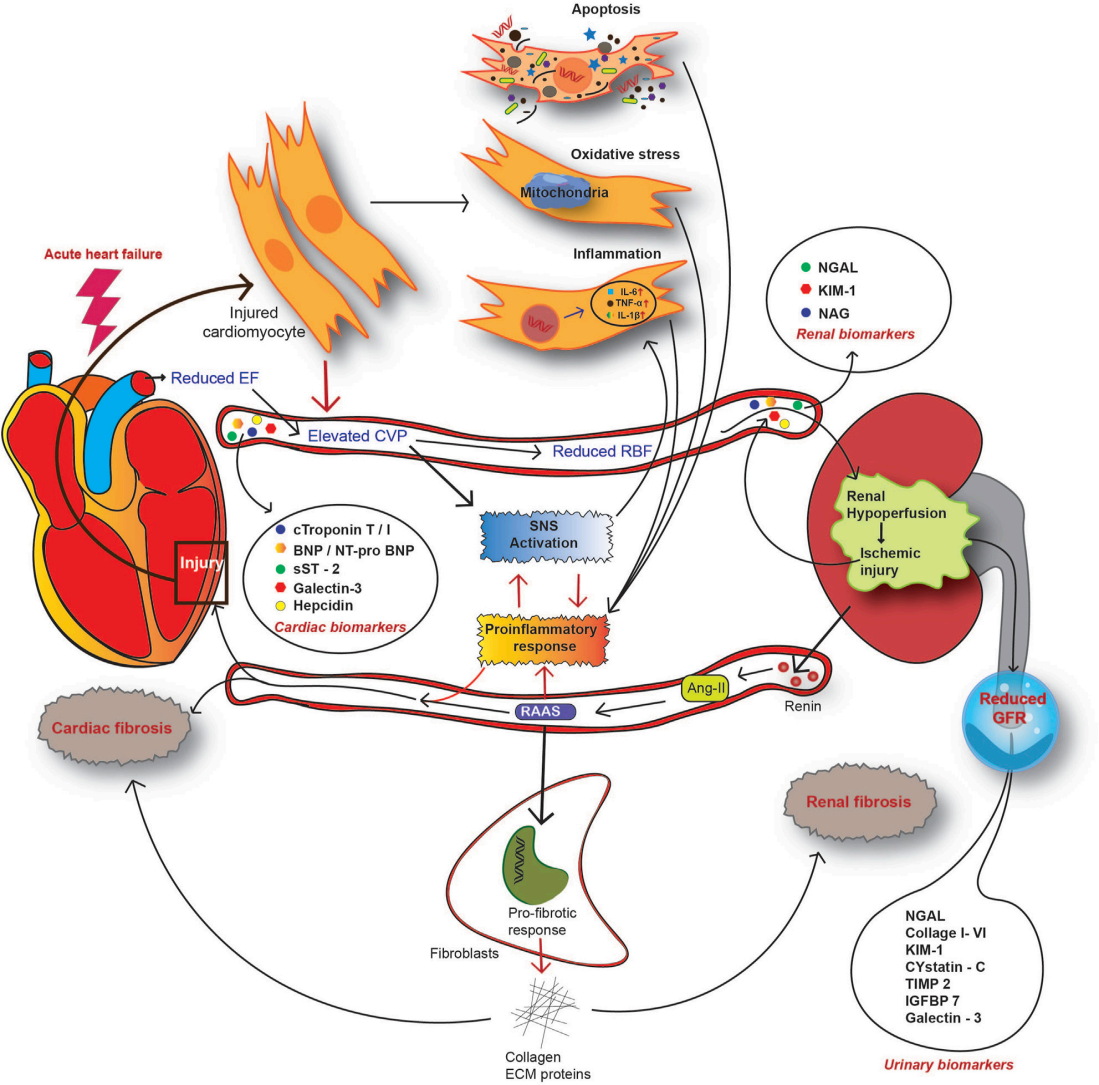
Overview of the pathophysiological mechanism of CRS in the setting of acute heart failure. In AHF patients, reduced CO causes renal hypoperfusion and subsequent activation of the renin-angiotensin-aldosterone system (RAAS). As a countermeasure, activation of the sympathetic nervous system (SNS) alongside RAAS enhances the systemic and local inflammatory response. Acute injury to both the heart and kidney releases a spectrum of molecules to circulation, designated as CRS biomarkers. CRS biomarkers from the heart include cardiac troponin (cTn) T or I, natriuretic peptide type-B (BNP), N-terminal proBNP, soluble ST2 (suppression of tumorigenicity 2), galectin-3, and hepcidin. Kidney injury biomarkers for CRS include serum NGAL, KIM-1, and NAG. A reduced GFR and impaired renal function contribute to the release of certain molecules into urine and this serves as an indicator of AKI. Such urinary biomarkers include urinary NGAL, collagen I to VI, KIM-1, cystatin-C, IGFBP7, galectin-3, and TIMP2.

Fundamental to a failing heart, activation of neurohormonal pathways is the physiologic response to the injury, and is strongly related to worsened outcomes in terms of mortality or morbidity (Rachwan et al., 2019). One of the pathways prototypical to CRS is the RAAS pathway, which is hyperactivated during the onset of acute CRS (Bongartz et al., 2005). RAAS is widely activated across various tissues, including the myocardium and kidney, and is pivotal in linking a damaged heart with worsening kidney functions (Rachwan et al., 2019; Biegus et al., 2021; Ma et al., 2022). Conventionally, systemic RAAS is critical for the maintenance of systemic or circulatory imbalances. In contrast, non-conventional axes of RAAS are a protective response to tissue injuries (Sharma et al., 2019; Holappa et al., 2020; Karimi et al., 2022). In ADHF, renal hypoperfusion leads to the activation of systemic RAAS response in the kidney. Specialized cells in the juxtaglomerular apparatus release renin*,* an angiotensin-converting enzyme, in response to decreased RBF and hyperactive SNS (Holappa et al., 2020). Increased plasma renin is associated with Ang-I–mediated release of epinephrine and nor-epinephrine (Ma et al., 2022). Plasma Ang-I is subsequently converted to angiotensin II, which is the crucial effector of the RAAS pathway (Verbrugge et al., 2015; Ma et al., 2022). Ang-II is a potent vasoconstrictor and can induce renal injury. On the other hand, Ang-II also causes oxidative stress and localized inflammatory responses. Activation of renal fibroblast to myofibroblast also causes local tissue inflammation, which further activates the so-called non-conventional or tissue RAAS axis (Long et al., 2004). Ang-II also stimulates aldosterone secretion as a product of the RAAS cascade, and under excessive RAAS activity, the plasma level of aldosterone increases (Weber and Brilla, 1991; Ma et al., 2022). Aldosterone, in accordance with Ang-II, stimulates local inflammation and fibrosis in the kidney and heart (Weber and Brilla, 1991; Long et al., 2004; Holappa et al., 2020). RAAS activation in an HF setting occurs as a compensatory mechanism in response to reduced CO and renal hypoperfusion (Takahama and Kitakaze, 2017). However, at increased plasma renin levels, persistent RAAS activation is also influenced by increased sodium retention and decreased excretion by tubular cells, leading to a reduced estimated eGFR and further worsening of renal injury in acute heart failure (Verbrugge et al., 2015). Continued overactivity of RAAS and SNS is counterproductive and leads to additional heart dysfunction due to heightened levels of oxidative stress, inflammation, and fibrotic ECM remodelling (Mentz and O'Connor, 2016). Aldosterone-mediated humoral response induces cardiac inflammation, remodelling, and fibrotic conditions, all leading to HF (Verbrugge et al., 2015; Takahama and Kitakaze, 2017; Ma et al., 2022). In the kidney, RAAS hyperactivation causes pathologic injury through vasoconstriction, inflammation, and fibrosis (Rajapakse et al., 2008; Takahama and Kitakaze, 2017). Moreover, aldosterone induces the production of reactive oxygen species (ROS) in tissues and further exacerbates the inflammatory response and cellular injury to both the heart and kidney (Ma et al., 2022). Thus, reduced CO, RBF, and renal glomerular-tubular feedback loop create a vicious cycle involving RAAS as a central player in hemodynamic disorders (summarized in Figure 4).

#### 3.1.2 Natriuretic peptide system (NPS)

The natriuretic peptide system (NPS) and RAAS function as opposing regulators in maintaining kidney and cardiovascular homeostasis (Peoples et al., 2019). The paracrine effect of RAAS and NPS is also associated with salt and water homeostasis through the kidney (Peoples et al., 2019; Shi et al., 2022). Three natriuretic peptides central to the NPS, atrial (ANP), and brain (BNP), as well as another non-natriuretic protein C-type natriuretic peptide (CNP), act as counter-regulators to the RAAS activities (Ronco et al., 2012a; Peoples et al., 2019). ANP and BNP act as diuretics that promote cardiac fibrosis in HF patients. Neprilysin is a widely distributed membrane-bound endopeptidase that degrades circulatory vasoactive peptides, such as ANP and BNP, and increases end-systolic blood pressure (Ames et al., 2019). However, contradictory to this effect, neprilysin also deactivates the RAAS pathway products Ang-I, Ang-II, and endothelin 1. Accordingly, patients with HF having upregulated soluble neprilysin and animal models of HF have shown enhanced renal neprilysin activity (Charniot et al., 2008; Junho et al., 2022). Moreover, elevated soluble neprilysin in serum is significantly correlated with adverse outcomes and is a good predictor of cardiovascular-related mortalities in HF patients (Charniot et al., 2008).

### 3.2 Non-hemodynamic coupling of the heart and kidney

#### 3.2.1 Oxidative stress

In healthy physiological conditions, ROS is produced in a balanced manner in every organ, including the heart and kidneys, for cellular functionalities (Milkovic et al., 2019; Peoples et al., 2019). However, under various pathological or physiological stress conditions, secondary and neurohormonal stimulation incurs a loss of oxidative reaction homeostasis, leading to increased production of ROS molecules by mitochondria, causing tissue damage (Shi et al., 2022). Impaired mitochondrial metabolism in the cardiomyocyte and kidney tubular cells is the final common pathway to tissue injury in CRS patients (Ronco et al., 2012a; Shi et al., 2022). Increased oxidative stress and RAAS activity in kidney tubular cells are responsible for water and sodium retention, leading to vasoconstriction and increased SNS activity (Ames et al., 2019). Hyperactive RAAS and SNS are central to the pathogenesis of acute CRS (Rangaswami et al., 2019).

In cardiomyocytes, xanthine oxidase, peroxisomes, xanthine, and NOS enzymes are the major source of ROS and/or RNS (reactive nitrogen species), whereas in the kidney NADPH oxidase (NOX_4_) is the primary source (Junho et al., 2022). A marked increase in the level of ROS markers is evident in AHF patients (Charniot et al., 2008). Imbalanced ROS and/or RNS levels directly impair the cardiomyocyte physiology and promote inflammatory responses (van der Pol et al., 2019). A gradual loss of cardiomyocytes results from increased oxidative stress, advancing maladaptive myocardial remodelling and fibrosis (Takimoto and Kass, 2007; van der Pol et al., 2019). Association of oxidative stress biomarkers, i.e., plasma aminothiols, cystine, and glutathione, has also shown increased death risk (Patel et al., 2016) and may also be potential biomarkers for CRS patients. Moreover, redox-regulatory protein TRX-1 is elevated in the urinary fraction in AKI and may also serve as an oxidative stress biomarker for kidney injury (Kasuno et al., 2014), but is yet to be tested in CRS patients. Oxidative stress plays a crucial role in developing AKI and is one of the primary aggravating stimuli (Tanaka et al., 2014). However, the functional effects of oxidative stress in CRS pathophysiology are unclear and the lack of suitable biomarkers for CRS patients makes it difficult to diagnose oxidative stress–induced myocardial or kidney injury.

#### 3.2.2 Inflammation

Myocardial ischemia (MI) is one of the primary causes of acute heart failure and is a major comorbidity condition in acute CRS patients (Table 2). Following ischemic injury, reduced circulation volume due to cardiac dysfunctionalities increases the activity of RAAS, SNS, and redox systems in the heart and mediates pathologic inflammation in acute heart failure patients (Riehle and Bauersachs, 2019). Following an ischemic injury in a rat model of MI, increased macrophage infiltration in the kidney and CCR2+-activated monocytes in circulation is evident, suggesting an inflammatory response against the injury (Cho et al., 2013). Angiotensin II (Ang-II) is primarily upregulated in the RAAS pathway and is a major effector in RAAS-mediated inflammatory responses in the NF-κB and AP-1 (Activator protein 1) pathways (Colombo et al., 2012). In acute CRS, hyperactivity of the RAAS axis and inflammation are widely reported (Colombo et al., 2012). Ang-II induction is associated with increased inflammatory cytokine (e.g., TNF-α, IL-6, and IL-1β) production by cardiomyocytes. Likewise, the inflammatory response in the kidney is also suggested to be mediated through the NF-κB pathway (Cho et al., 2013). Moreover, C-reactive protein (CRP), an acute phase inflammatory mediator marker upregulated in AKI, is independently correlated with higher mortality in ADHF patients (Colombo et al., 2012; Kumar et al., 2019). Recently, an association between inflammatory markers and CRS has been explored in HF patients with renal failure (Jensen et al., 2010; Colombo et al., 2012). Such inflammatory mediators associated with AHF or AKI or directly with CRS supposedly play active roles in acute CRS pathogenesis and have prognostic value in CRS patients.

**TABLE 2:** Common aetiologies, gender (%), CRS prevalence, and biomarker levels in patients hospitalised for symptomatic AHF. Ischaemic heart disease (IHD), acute heart failure (AHF), acute decompensated heart failure (ADHF), diabetes mellitus (DM), acute kidney injury (AKI), chronic kidney disease (CKD), coronary artery disease (CAD), myocardial ischemia (MI), heart failure (HF), and acute coronary syndrome (ACS) are the most common comorbidities in acute CRS patients worldwide. Values are the mean ± SD (standard deviation).

Primary setting	Aetiology of AHF	Gender prevalence	AKI/WRF	Biomarkers and levels (mean ± SD)	Ref
ADHF	Diabetes	—	58 out of 145 patients developed AKI (40%)	**sCr** 1.9 ± 0.9 mg/dL	Mullens et al. (2009)
	Hypertension	—	—	**BUN** 58 ± 25 mg/dL	—
	Hyperlipidaemia	—	—	**GFR** 48 ± 19 mL/min/1.73 m^2^	—
	—	—	—	**BNP** 1,559 ± 1,340 pg/mL	—
ADHF	Hypertension (85.6%)	60.9% male	174 out of 705 patients had WRF (15%)	**sCr** > 1.4 mg/dL	Nunez et al. (2017)
	Diabetes (56.3%)	—	—	**eGFR** 33 mL/min/1.73 m^2^	—
	—	—	—	**Urea** 92 ± 35 mg/dL hs-TnT 49 ng/L	—
	—	—	—	Haemoglobin 11.4 ± 1.8 g/dL	—
	—	—	—	**LVEF** 47.9% ± 15.8%	—
	—	—	—	**Left atrium diameter** 43.5 ± 7.3 mm	—
Children hospitalized for symptomatic heart failure	Dilated cardiomyopathy (52%)	55% male	35 out of 73 were admitted (49%)	**sCr** > 1.0 ± 0.7 mg/dL	Price et al. (2008)
—	ADHF (26%)	—	—	**BUN** 25 ± 18 mg/dL	—
—	Ischemic cardiomyopathy (10%)	—	—	**BNP** 2480 ± 1,465 pg/mL	—
—	Myocarditis (12%)	—	—	—	—
ADHF	Ischemic heart disease in 56% of progressive AKI vs. 55% in non-progressive AKI.	52% of males developed progressive AKI, while 66.3% of males were in the non-progressive AKI group	213 out of 732 patients developed AKI (29%)	**sCr** >1.5 ± 0.8 mg/dL in progressive AKI compared to >1.6 ± 0.8 mg/dL in non-progressive AKI.	Chen et al. (2016)
	—	—	—	**NT-proBNP** - 9,000 pg/mL in progressive AKI compared to 6,647 in non-progressive AKI.	—
	—	—	—	**Haemoglobin** 10.8 ± 2.4 g/dL in progressive AKI compared to 11.8 ± 2.6 g/dL in non-progressive AKI.	—
	—	—	—	**eGFR** 59.1 ± 28.3 mL/min per 1.73 m^2^ in progressive AKI compared to 63.4 ± 24.0 mL/min/1.73 m^2^	—
AHF/ADHF	Arterial hypertension (70.8%)	51.4% male	34.53%	**sCr (D1)** level of 2.44 [1.47–4.09] mg/dL and **eGFR**_**CKDEPID1**_ 22 [13–44] mL/min per 1.73 m^2^	Phan Thai et al. (2020)
	Diabetes mellitus (42.6%)		48 out of 139 developed CRS type 1	**sCr (D3)** level of 2.84 [1.38–4.8] mg/dL and **eGFR**_**CKDEPID3**_ 19.5 [11–47.5] mL/min per 1.73m^2^	—
	HF (35.4%)	—	—	**Urea** 12.67 [8.51–19.27] mmol/L	—
	Acute MI (40.9%)	—	—	**NGAL** 506.49 [322.51–591.80] ng/mL	—
	ADHF (50%)	—	—	**NT-proBNP** 20131 [6,350–35,000] pg/mL	—
AHF	HF (46%)	44% male	46 out of 292 patients developed AKI (15.75%)	**sCr** 0.98 (0.87–1.48) mg/dL	Lassus et al. (2010)
	Coronary artery disease (70%)	—	—	**Cystatin C** 1.44 (1.16–1.91) mg/L	—
	MI (30%)	—	—	**NT-proBNP**—7,379 (3,517–16,883) pg/mL	—
	Hypertension (63%)	—	—	—	—
	Diabetes (37%)	—	—	—	—
	Chronic atrial fibrillation (26%)	—	—	—	—
Children undergoing cardiopulmonary bypass surgery	—	65% male	20 out of 71 developed AKI (28%)	**sCr** change is 99%	Mishra et al. (2005)
Acute MI	Hypertension (65.9%)	63.4% male	410 out of 1,371 patients developed AKI (29.9%)	**Baseline eGFR** is 52.5 mL/min per 1.73m^2^	Sun et al. (2016)
	Hyperuricemia (31.5%)	—	—	**Haemoglobin** 127.5 g/L	
	Diabetes (38.5%)	—	—	**LVEF** 55%	
	Acute infection (34.9%)	—	—	—	
Acute HF syndrome (AHFS)	Ischemic etiology of heart failure (70.19%)	71.1% male	115 out of 448 patients (25.7%)	**Baseline sCr** is 1.7 ± 0.7 mg/dL in transient WRF and 2.1 ± 1.4 mg/dL in persistent WRF	Aronson and Burger (2010)
	Diabetes mellitus (56.7%)	—	—	**Baseline eGFR** is 49 ± 23 mL/min per 1.73 m^2^ in transient and 42 ± 24 mL/min per 1.73 m^2^ in persistent WRF	—
	Atrial fibrillation (41.3%)	—	—	—	—
HF/ADHF patients	Atrial fibrillation (58%)	71.7% male	244 out of 635 patients (38.3%)	**Baseline eGFR,** 65.3 ± 26.5 mL/min per 1.73 m^2^	Roy et al. (2013)
—	Diabetes (30.5%)	—	—	—	—
ADHF	Arterial hypertension (71%)	61% male*	136 out of 657 patients (20.7%)	**Urea** 12.6 [8.6–22.1] mmol/L **sCr** 115 [86–156] mmol/L	Breidthardt et al. (2011)
	Heart failure (61%)	—	—	**GFR** 49 [21–68] mL/min per 1.73 m^2^	—
	CAD (53%)	—	—	**BNP** 1071 (709–1,714) pg/mL	—
	Diabetes mellitus (36%)	—	—	—	—
	CKD (58%)	—	—	—	—
ACS	Hypertension (60.5%)	53.5% male	36 out of 236 patients (15.2%)	**BUN** 26.75 ± 15.72 mg/dL **sCr** 1.23 ± 0.68 1.98 ± 1.41	Eren et al. (2012)
	Diabetes mellitus (39.4%)	—	—	**eGFR** 66.69 ± 26.67 mL/min per 1.73 m^2^	—
	CKD (46.5%)	—	—	**Haemoglobin** 12.39 ± 2.50 g/dL	—
	—	—	—	**EF (%)** 46.11 ± 9.93	—
ADHF	—	—	35 out of 53 patients (66%)	**BUN** 48.22 ± 26.53 mg/dL **sCr** 1.98 ± 1.41 mg/dL	Eren et al. (2012)
	—	—	—	**eGFR** 37.62 ± 18.35 mL/min per 1.73 m^2^	
	—	—	—	**Haemoglobin** 11.24 ± 2.03 g/dL	
	—	—	—	**EF (%)** 37.94 ± 12.00	
ADHF	IHD (32.3%)	56.7% male	275 out of 376 (73.1%)	**sCr** 1.33 ± 0.79 mg/dL	Hata et al. (2010)
	Valvular heart disease (32.3%)	—	—	**BNP** 1110 ± 1,203 mg/mL	
	Hypertensive heart disease (17.4%)	—	—	—	
	Cardiomyopathy (13.4%)	—	—	—	
AHF	Hypertension (65%)	52.7% male	391 out of 614 (63.6%)	**eGFR** 56.4 ± 27.0 mL/min per 1.73 m^2^	Li et al. (2014)
	Diabetes (26.6%)	—	—	**Hb** 112 ± 26 g/L	
	IHD (27.2%)	—	—	—	
AHF	Hypertension (50%)	60% male	152 out of 416 (36.5%)	**eGFR** 59 ± 41 mL/min per 1.73 m^2^	Logeart et al. (2008)
	Diabetes (30%)	—	—	**Hb** 12.1 ± 1.7 g/dL	
	Ischemic cardiopathy (62%)	—	—	—	
AHF	Coronary artery disease (61%)	68% male	107 out of 318 (33.6%)	**QRS duration** 136 ± 41 ms (+9 ms increase)	Metra et al. (2008)
	Idiopathic cardiomyopathy (34%)	—	—	**sCr** is 1.45 ± 0.67 mg/dL (peak 2.26 ± 1.06 mg/dL)	
	—	—	—	**BUN** 69 ± 37 mg/dL (peak 102 ± 61 mg/dL)	
	—	—	—	**GFR** 62 ± 37 mL/min, decreasing to lowest at 36 ± 10 mL/min	
	—	—	—	**LVEF (%)** decreased	
Acute ST elevation MI	Hypertension (52%)	72% male	98 out of 1,019 patients (9.6%)	**BUN** 26 [16–31] mg/dL **sCr** 1.2 [1.0–1.6] mg/dL	Goldberg et al. (2005)
	Diabetes (20%)	—	—	**GFR** 59 [42–80] mL/min	
ACS	Hypertension (65%)	74% male	409 out of 3,210 (12.7%)	**sCr** is 1.6 ± 1 mg/dL	Marenzi et al. (2013)
	Hyperlipidaemia (36%)	—	—	**eGFR** 53 ± 23 mL/min per 1.73 m^2^	
	MI (35%)	—	—	**LVEF (%)**—42 ± 14	
ADHF	Ischemia 37.7%	95 (54.6%) males	Early AKI 174 out of 625 (27.8%)	**BUN** 32.6 ± 21.5 mg/dL	Shirakabe et al. (2013)
	—	—	—	**sCr** 1.57 ± 1.02 mg/dL	
	—	—	—	**BNP** 1,230.7 ± 1,305.8 pg/mL	
	Ischemia 42.9%	104 (61.2%) males	Late AKI 170 out of 625 (27.2%)	**BUN** 23.5 ± 10.4 mg/dL **sCr** 1.21 ± 0.68 mg/dL	
	—	—	—	**BNP** 983.0 ± 1,376.9 pg/mL	
AHF/ADHF (total 419 admitted)	Hypertension 50%	67.7% male	78.3% survived (328)	**HR (bpm)** 86.3 ± 21.1	Wang et al. (2020)
66.1% male	Diabetes mellitus 20.7%	—	—	**Hb** 13.5 ± 2.1 g/dL	
—	Atrial fibrillation 36%	—	—	**NT-proBNP** 1,091.5–4,643.5 (2035.0) pg/mL	
—	—	—	—	**BUN** 5.6–8.5 (6.8) mM	
—	—	—	—	**GFR** 63.9–97.6 (79.3) mL/min per 1.73 m^2^	
—	—	—	—	**Uric acid** 463.5 ± 148.8 μM	
—	—	—	—	**QRS duration** 128.4 ± 40.7 ms	
—	Hypertension 42.9%	60.4% male	21.7% died (91)	**HR (bpm)** 83.5 ± 21.3	Wang et al. (2020)
—	Diabetes mellitus 25.3%	—	—	**Hb** 12.6 ± 2.2 g/dL	
—	Atrial fibrillation 37.4%	—	—	**NT-proBNP** 1,662.3–7,972.8 (2,926.0) pg/mL	
—	—	—	—	**BUN** 6.5–11.6 (9.1) mM	
—	—	—	—	**GFR** 42.5–91.1 (62.3) mL/min per 1.73 m^2^	
—	—	—	—	**Uric acid** 537.4 ± 212.0 μM	
—	—	—	—	**QRS duration** 133.9 ± 38.3 ms	

#### 3.2.3 Apoptosis

Following MI, kidney tubular cell apoptosis is evident in animal models (Cho et al., 2013). Inflammation-mediated apoptosis is thought to be involved in kidney damage in AKI (Virzi et al., 2012). Incubation with plasma from CRS type I patients strongly induces apoptotic death in monocytes, correlating with the suggested hypotheses of Cho et al. (2013) (Virzi et al., 2012; Cho et al., 2013). Moreover, kidney cell apoptosis is associated with upregulated TNF-α and IL-6 levels (Cho et al., 2013), pointing towards the role of inflammation-mediated pathological apoptosis–induced injury in AKI.

## 4 Molecular pathophysiology of CRS

Recently, discoveries of novel proteomic biomarkers and miRNAs associated with CRS have allowed new molecular mechanisms to be described. In the acute cardiorenal nexus, inflammatory responses are exaggerated to alleviate primary myocardial injury, which further aggravates injury to both the heart and kidney. CXCR4 is a G protein-coupled chemokine receptor associated with the trafficking of immune cells to injured organs (Chu et al., 2011; Yuan et al., 2015). CXCR4 is the cognate receptor for SDF-1 and helps in aggravating inflammation-induced cardiorenal fibrosis (Chu et al., 2011). In acute ST-segment elevated MI patients, CXCR4 signals after cardiac injury predict adverse outcomes in such patients (Werner et al., 2021). Prolonged CXCR4 expression in renal cell types is also found to be associated with injury-mediated fibrotic response (Yuan et al., 2015; Werner et al., 2021). Hypertension is one of the most common comorbidity aetiologies associated with acute CRS and is associated with neurohormonal RAAS hyperactivation, inflammatory responses, and CRS progression (Chu et al., 2011).

**FIGURE 5:**
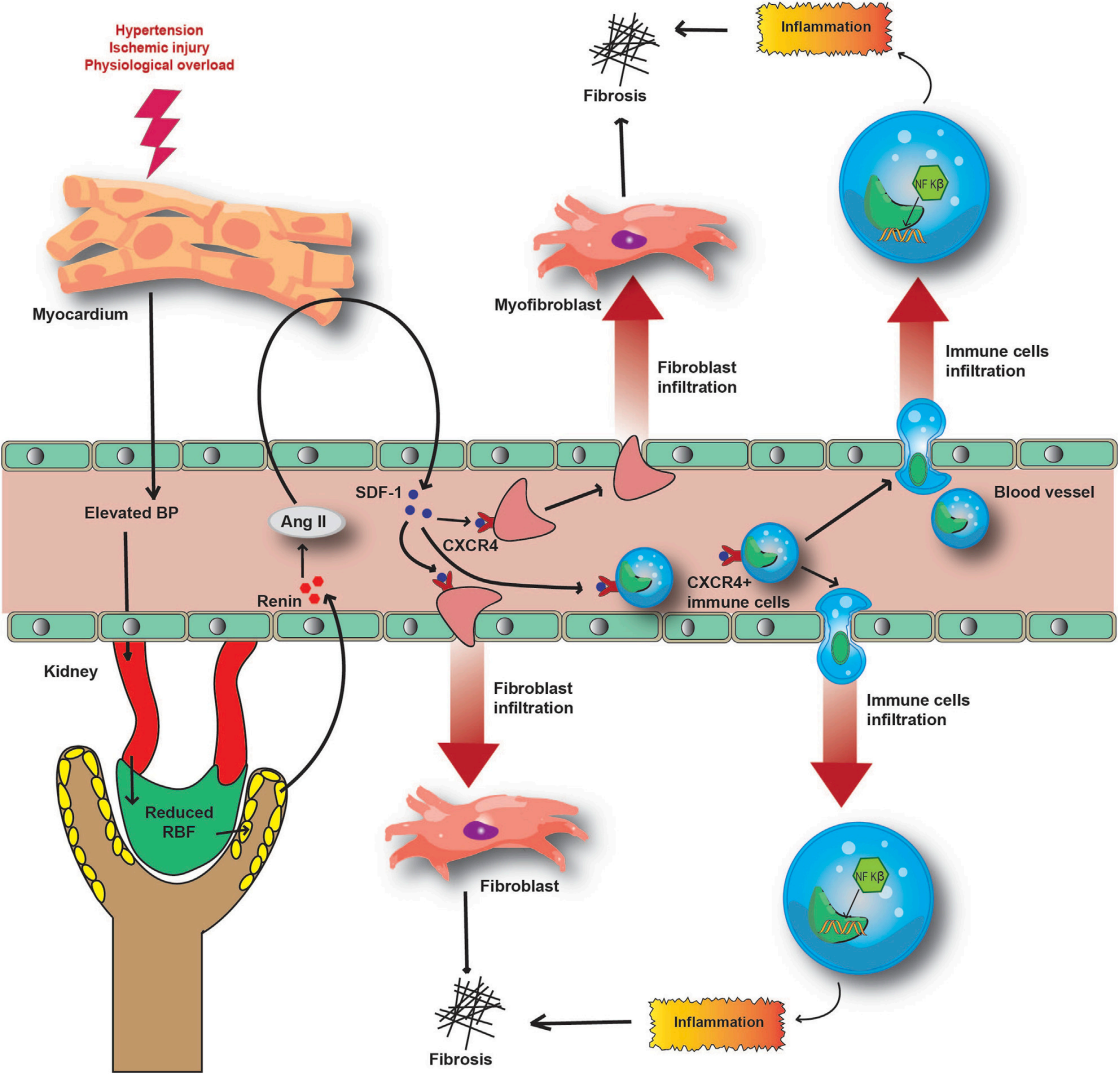
Molecular mechanism underlying the inflammatory and fibrotic response in CRS pathophysiology. Acute injury to the heart and subsequently to the kidney causes the release of SDF-1 to circulation. Hyperactivation of RAAS induces the SDF-1- CXCR4 interaction and the infiltration of immune and fibrotic cells to local inflammatory sites. This further aggravates inflammation and fibrosis in damaged organs, reducing their functionalities.

Acute inflammation is strongly linked with cardiorenal fibrosis. Angiotensin II-mediated activation of stromal-derived factor (SDF)-1 is associated with the infiltration of pro-fibrotic immune cells to the site of local inflammation (Chu et al., 2011; Van Linthout et al., 2014). Moreover, neurohormonal or Ang-II stimulation of cardiomyocytes is associated with overexpression of SDF-1 (Chu et al., 2010). Increased levels of SDF-1 interact with CXCR4 and promote fibroblast infiltration during cardiorenal fibrosis (Chu et al., 2010; Chu et al., 2011). The acute inflammatory response in the myocardium is responsible for the release of inflammatory cytokines and chemokines such as TNF-α, IL-6, IL-18, and MCP-1 by the infiltered immune cells (Virzi et al., 2018; Jin et al., 2021). Higher circulating pro-inflammatory cytokine levels are evident in acute CRS patient samples (Virzi et al., 2018). Overexpression of TNF-α, MCP-1, VCAM-1, and ICAM-1 in the renal tissue in CRS is directed towards an inflammatory response in the kidney (Jin et al., 2021). This suggests that aggravated local and systemic inflammatory responses participate in the intra-organ crosstalk, lying at the centre of the pathogenesis of CRS (Jin et al., 2021). In combination with inflammation, a coalition of RAAS and angiotensin type −1 (AT-1) receptor-mediated NF-κB signalling has recently been described (Li and Zhuo, 2008). Even though the distinct mechanism is yet to be studied, pro-inflammatory molecules have emerged as potential marker candidates, due to the association between inflammation and CRS pathophysiology (Figure 5).

## 5 Clinically available biomarkers for acute CRS

### 5.1 Serum creatinine

An increased serum creatinine (sCr) level is one of the standard parameters for determining acute CRS compared to other types (I–V) (Gai et al., 2010). AKI is defined by the rise of creatinine in serum, and, even with certain non-specificity, it is a gold standard as of now to predict and diagnose kidney injury (Grynberg et al., 2017). In patients undergoing cardiac surgery, AKI onset is predictable through the rise in sCr from baseline to post-operative day 1 and is associated with longer hospitalisation and significantly increased morbidity (Zappitelli et al., 2009; Ho et al., 2012; Grynberg et al., 2017). The normal level of serum creatinine ranges from 0.6 to 1.2 mg/dL in males and from 0.5 to 1.1 mg/dL in females (Hosten et al., 1990). AKI worsening in hospitalized ADHF patients is strongly correlated with serum creatinine levels; 36% of patients with sCr levels >2.0 mg/dL have worsened AKI after admission (Chen et al., 2016). In the Indian population, sCr is significantly upregulated in CRS patients who have worse clinical outcomes (Reddy et al., 2020).

#### 5.1.1 Limitations

Despite being a significant biomarker of WRF, sCr fails to accurately assess the degree of kidney dysfunctionalities during acute injury-mediated changes (Bellomo et al., 2004). The sCr level is insensitive to minor to moderate changes in GFR and only increases after more than a moderate change in GFR (Murty et al., 2013). Moreover, creatinine is produced in the liver and the sCr levels can be influenced by conditions such as fever, liver-disease, or ageing, which interfere in accurate diagnosis (Bellomo et al., 2004). In the Indian population, sCr is studied as a diagnostic parameter, but considering all these limitations, sCr is less reliable in detecting early AKI in CRS patients (Reddy et al., 2020).

### 5.2 Blood urea nitrogen (BUN)

Blood urea nitrogen (BUN) is typically used to diagnose AKI in combination with sCr (Edelstein, 2008). Urea in blood is excreted by cells as a metabolic by-product and is freely filtered through glomerulus (Seki et al., 2019). BUN in serum is the nitrogen fraction of urea, which normally ranges between 5 and 20 mg/dL (1.8–7.2 mmol urea/L) (Hosten et al., 1990). An increase in BUN levels indicates declining renal functionalities (Seki et al., 2019). In acute CRS patients, hyper-activation of RAAS, SNS, and the neurohormonal system causes upregulation in BUN and acts as a predictor for kidney functionalities (Qian et al., 2019). An elevated BUN/sCr ratio can predict declining GFR in acute CRS patients.

#### 5.2.1 Limitations

The broad reference range of BUN makes it suboptimal for diagnosing acute CRS. Independent of kidney functionalities, and being a metabolic by-product, BUN is influenced by several nonrenal factors such as dietary protein intake, protein catabolism, high-dose steroid therapies, hepatic urea synthesis, etc., (Yu, 2020). A significantly higher BUN level is strongly associated with unfavourable outcomes among CRS patients in India (Reddy et al., 2020).

### 5.3 Cystatin C

Cystatin C (CysC) is a 13.3 kDa cysteine protease inhibitor protein synthesized by all nucleated cells. Serum CysC is freely filtered through the glomerulus into urine and is thus considered a parameter in assessing GFR to evaluate renal functioning. Unlike serum creatinine, once in the glomerular filtrate, CysC is not further reabsorbed or secreted directly into the urinary fraction by tubular cells (Laterza et al., 2002). This makes it a more accurate endogenous surrogate marker for GFR than sCr. CysC measurement in ACS patients has improved risk stratification early on admission, making it a promising biomarker for acute CRS (Jernberg et al., 2004).

#### 5.3.1 Limitations

Although CysC is a more accurate predictor of GFR in AKI compared to sCr, factors such as high body fat mass, BMI, diabetes, inflammation, and other disorders like thyroid affect serum CysC levels (Muntner et al., 2008; de Vries and Rabelink, 2013; Gonzalez et al., 2022). Moreover, it has been reported that patients undergoing cardiac surgery had minimal difference in CysC levels (Shlipak et al., 2013). The serum CysC level is affected in AHF patients without renal impairment (Naruse et al., 2009), making it unreliable in diagnosing acute CRS patients. Moreover, older age, male gender, smoking, and higher CRP levels independently regulate the CysC level, irrespective of AKI (Knight et al., 2004). Although CysC is significantly correlated with eGFR and renal dysfunctionalities in different age groups in India, there is still a gap in terms of real world evaluation in CRS patient samples (Kumaresan and Giri, 2011).

### 5.4 Glomerular filtration rate

The glomerular filtration rate (GFR) is an index of kidney function, measuring the plasma flowing through it during a certain period (e.g., per minute). A decrease in GFR below the baseline of 60 mL/min per 1.73 m^2^ (Mullens et al., 2009) indicates dysfunctionalities in kidney tubules. Normal or mild renal insufficiency was defined at a GFR of 60 mL/min/1.73 m^2^ (Mullens et al., 2009). In CRS patients, venous congestion causes under perfusion of the kidney. Kidney hypoperfusion is responsible for impaired GFR and is a strong risk factor for AKI or WRF. Myogenic control over GFR is intrinsically influenced by activated RAAS, SNS, and ROS/NO imbalance during CRS, and causes arterial underfilling in a failing or ischemic heart (Viswanathan and Gilbert, 2010). Moreover hysterical RAAS and neurohumoral over-activation causes vasoconstriction and decreased RBF (Ljungman et al., 1990). Decreased RBF induces renal ischemia in the heart failure setting (Viswanathan and Gilbert, 2010; Rangaswami et al., 2019). Evaluating eGFR at admission somewhat predicts AKI in HF patients. Declining GFR in CRS patients is correlated with in-hospital worsened outcome and increased mortality (Wang et al., 2020). A single centred prospective study in India associated reduced eGFR with non-favourable outcome in-hospital as well as in discharged CRS patients (Reddy et al., 2020).

#### 5.4.1 Limitations

Decongestive measure in AHF patients may complicate proper assessment of eGFR, thus limiting the diagnosis of AKI (Rangaswami et al., 2019). GFR in AKI is measured by evaluating BUN and sCr, which do not always correlate with GFR (Molitoris, 2012). This limits the early detection of AKI due to a misleading GFR calculation. Moreover, inaccuracy in eGFR calculation causes incorrect prediction in respect to worsening AKI (Kirwan et al., 2013); thus, a new standard needs to be set using more accurate biomarkers. Although the eGFR rate is associated with worsened outcomes in CRS patients in India, it needs to be evaluated in a larger cohort of CRS patients (Prothasis et al., 2020).

### 5.5 Serum and/or urine neutrophil gelatinase-associated lipocalin (NGAL)

NGAL, also known as lipocalin-2 or siderocalin, is a 25 kDa small, glycosylated protein belonging to the lipocalin superfamily, and was initially described to be secreted from granular neutrophils upon specific stimulus (Cowland and Borregaard, 1997; Kokkoris et al., 2012). Human NGAL is a 25-kDa immunoreactive protein secreted by the neutrophils (Bolignano et al., 2008). Kidney tubular cells also secrete NGAL as an acute phase factor to immediately signal a condition on injury (Mishra et al., 2003; Bolignano et al., 2008). NGAL is also secreted at a basal level by cardiomyocytes, kidney tubular cells, and other tissue cells (Cowland and Borregaard, 1997). In ischemic kidney injury or renal toxicity, NGAL is overexpressed in kidney tubular cells as an early post-ischemic response (Kokkoris et al., 2012; Rangaswami et al., 2019; Phan, 2021). In patients with cardiac surgery, serum and urine NGAL levels increased 10-fold or more and were an early biomarker for the onset of AKI (Palazzuoli et al., 2014). Patients hospitalized with ADHF and having higher than baseline NGAL levels were correlated with AKI and mortality (Macdonald et al., 2012). In contrast to traditional sCr, increased NGAL levels are detected 1–3 h after surgery in such patients and are also supported by animal model data (Mishra et al., 2003; Kokkoris et al., 2012). AKI or WRF are diagnosed by RIFLE in 25%–40% of patients admitted with ADHF (Ronco et al., 2012b; Palazzuoli et al., 2014). NGAL measurement at admission is suggested for the early detection of WRF in acute CRS patients, and NGAL levels above 130–170 ng/mL are correlated with adverse clinical outcomes (Alvelos et al., 2011; Palazzuoli et al., 2014).

The relative abundance of monomeric NGAL in AKI patient plasma samples is principally due to secretion by stressed kidney epithelial cells as monomers, which fail to further dimerize. A 100-fold rise in urinary and a 20-fold rise in plasma NGAL levels have allowed prediction of AKI in AHF patients well before any significant change in sCr levels (Martensson and Bellomo, 2014).

#### 5.5.1 Limitations

Although NGAL is widely accepted as an early biomarker for AKI in CRS patients, differences in cut off values and responses in individuals limit its application. However, high-sensitivity assays for plasma NGAL with a series of measurements can increase the sensitivity of the diagnosis of AKI in acute CRS (Ronco et al., 2012b). Combined plasma NGAL, Cystatin C, and NT-proBNP values above basal levels could significantly improve the predictive accuracy of the diagnosis of CRS in ADHF or AHF patients (Phan, 2021; Song et al., 2021). In respect to the Indian CRS cohort, NGAL has not yet been evaluated in any clinical studies, limiting the diagnostic ability of such proteomic markers.

### 5.6 Brain natriuretic peptide

BNP (B-type natriuretic peptide) is predominantly synthesized by LV cardiomyocytes as a protective mechanism against pressure or volume overload (Cao et al., 2019; Okamoto et al., 2019). BNP mimics ventricular stress by decreasing the kidney tubule vascular resistance and increasing GFR (Okamoto et al., 2019; Sarohi et al., 2022a). However, hyperactivation of RAAS and SNS inhibit the BNP activity and worsens the condition (Okamoto et al., 2019). Human BNP is a 134 amino acid peptide that, under pathological conditions, is rapidly cleaved to form a 108 amino acid sequence, namely, pro-BNP. Pro-BNP is cleaved by furin/corin/proNP convertase to release BNP and NT-pro BNP to circulation (Cao et al., 2019). In patients with AHF or MI, a transient rise occurs in the circulating N-terminal pro-BNP and pro-BNP levels (Breidthardt et al., 2011; Phan Thai et al., 2020). Patients with worsened in-hospital outcomes have elevated levels of plasma pro-BNP compared to surviving CRS patients (Wang et al., 2020). In healthy individuals, the circulating baseline level ranges from 21 to 281 pg/mL in males and 51–240 pg/mL in females (Welsh et al., 2022). An elevated baseline NT-proBNP level is profoundly associated with acute CRS (Sabatine et al., 2004; Chen et al., 2016). Moreover, a drop in serum NT-proBNP increases the chance of survival and is associated with fewer re-hospitalisation events (Gembillo et al., 2021).

#### 5.6.1 Limitations

The circulating NT-proBNP level increases with age and corresponds to age-related health conditions. In elderly individuals with no cardiovascular complications, the NT-proBNP level can hover at a much higher level than the baseline (Welsh et al., 2022), thus limiting its application as a unique protein biomarker for diagnosing CRS. Similar to NGAL, BNP/pro-BNP or NT-pro BNP are yet to be evaluated in clinical CRS patient samples in India.

### 5.7 Other biomarkers in use

Biomarker investigation across all CRS types has revealed a number of promising biomarkers; however, these are yet to be studied significantly. The serum hepcidin level is presented as being associated with relative thickening of the LV wall (Kim, 2020).

Apart from clinical investigations, a handful of protein biomarkers are repeatedly studied in acute and chronic CRS. Kidney Injury molecule 1 (KIM-1), sST2, Galectin-3, N-acetyl-κ-d glycosaminidase (NAG), liver-type Fatty acid-binding proteins (L-FABP), insulin-like growth factor–binding protein 7 (IGFBP7), and tissue inhibitor of metalloproteinase 2 (TIMP2) are some recent biomarkers that have been studied in terms of CRS (Tan and Sethi, 2014; Rangaswami et al., 2019; Goffredo et al., 2021).

#### 5.7.1 KIM-1

KIM-1 is a transmembrane receptor (type-1) glycoprotein, dispersedly distributed in the epithelial cell surface (Song et al., 2019). It is also found in proximal tubular epithelial cells of the kidney in acute injury conditions (Song et al., 2019). In AKI patients, kidney tubular epithelial cells express KIM-1, which, after injury, are present in the urinary compartment, leading to detection of KIM-1 in urine (Zhang et al., 2007). Recent studies have shown that upregulated KIM-1 expression in chronic CRS patients is in alignment with NGAL (Kaddourah et al., 2016). In AKI, limited and sudden elevation of urinary KIM-1 in AKI makes it an advantageous candidate as a potential biomarker for acute CRS diagnosis (Medic et al., 2015). However, urinary KIM-1 levels were found not to be statistically significant in prognosing acute CRS (Atici et al., 2019). On the other hand, in a recent multicentre prospective study, Chen et al. found KIM-1 to be a valuable predictor of AKI in ADHF patients (Chen et al., 2016). Moreover, the Plasm KIM-1 level was found to be higher in cardiac surgery patients having AKI compared to that in patients without AKI and this was validated against animal smodels (Sabbisetti et al., 2014).

#### 5.7.2 Galectin-3

Galectin-3 (Gal-3) is a 30 kDa β-galactoside-binding lectin involved in cell-cell and cell-ECM interactions. Gal-3 is secreted by injured and inflammatory cells into the serum and is subsequently also found in urine (Hara et al., 2020). In acute heart failure, it has already been described and validated as an early diagnostic biomarker (Hara et al., 2020). Moreover, reduced GFR affects the Gal-3 level in HF patients compared to that in heart dysfunction patients (Podzolkov et al., 2022).

#### 5.7.3 NAG

NAG is a 130 kDa lysosomal enzyme secreted upon the exocytosis or degradation of cells in healthy tissues. Due to its high molecular weight it is not filtered through the glomerulus and its levels are generally low in the urinary compartment (Hashimoto et al., 1995). However, in an injury event or lesion to the nephron, the urinary level of NAG increases abruptly and this signifies AKI(140). The NAG level is found to be upregulated in both AKI and hypertensive patients (Hashimoto et al., 1995; Goffredo et al., 2021) and plays a possible role in the progression of acute CRS.

#### 5.7.4 TIMP-2 and IGFBP7

IGFBP7 (insulin-like growth factor–binding protein 7) and TIMP2 (tissue inhibitor of metalloproteinase 2) are biomarkers of cell cycle arrest. Studies have described upregulated urinary IGFBP7 and TIMP2 levels in early AKI patients, in alignment with NGAL (Sakyi et al., 2021).

Other than these, cardiac Troponin T (cTnT) and cTnI are highly sensitive prognostic and diagnostic biomarkers of AHF/ADHF, which is a potential biomarker of acute CRS. Elevated cardiac troponins are correlated with declining GFR and associated with increased mortality in CKD patients (Colbert et al., 2015; Rangaswami et al., 2019) and needs to be studied in acute CRS also. Inflammatory cytokine tumor necrosis factor alpha-α (TNFα) is reported to be elevated in a cohort of AHF patients and is associated with increased mortality (Dunlay et al., 2008). TNFα and other inflammatory biomarkers, such as IL-6, are potential biomarkers and may be evaluated in acute CRS patients. To date, most clinical studies in India diagnose CRS based on echocardiographic parameters (Tandon et al., 2013; Reddy et al., 2020). Very few biomarkers of CRS are evaluated in the Indian population, limiting early detection of AKI in future CRS patients.

## 6 Omics in acute CRS

The acute form is highly prevalent among all five CRS subtypes and is associated with increased mortality/severity in AHF/ADHF/ACS and cardiac surgery patients. To date, clinical studies have evaluated diagnostic-based biomarkers. Recently, omics-based approaches were used in a number of studies on urinary or plasma counterparts in CRS patients. Recently, a case–control study by Petra et al. performed a large scale urinary peptidome analysis in CRS patients and analysed peptides and proteins in urine (Petra et al., 2021). The study described 30 protein precursors detected in CRS patients only, which are absent in either HF or CKD patients. This includes several cardiac and renal ECM proteins, including COL4A2, COL4A4, and COL6A5. Other proteins, such as ROBO1, HUWE1, and CD14, are associated with fibrosis and dysfunction of the heart and kidney and are uniquely found in CRS urinary peptidome (Zhu et al., 2020; Liu et al., 2021; Petra et al., 2021; Sarohi et al., 2022b). Moreover, immune response–associated proteins, such as CD14, CD99, and IRF-6, are correlated with a hyperactivated immune response in CRS patients (Rangaswami et al., 2019; Liu et al., 2021). In a rat model of HF, renal proteome analysis showed upregulation of advanced glycosylation product-specific receptor (RAGE), renal angiotensin converting enzyme (ACE), and angiotensin II (Melenovsky et al., 2018). RAGE is a DNA sensor protein that is linked to damage associated molecular pattern (DAMP)–mediated inflammation in HF. This suggests a possible crosstalk between RAGE-immune signalling and ACE-mediated Ang-II signalling in acute CRS progression (Melenovsky et al., 2018). Upregulation of periostin, collagen VI, gal-3, and so on in the HF kidney proteome suggests fibrosis in HF models. Gal-3 is a well-studied HF biomarker associated with worsened outcomes in HF and CKD patients (Melenovsky et al., 2018; Sarohi et al., 2022b).

### 6.1 miRNA as acute CRS biomarker

miRNAs are endogenously conserved non-coding RNA molecules, associated with post-transcriptional gene regulation (Huang et al., 2020). In humans, thousands of such mRNA have been described and hundreds of them have been reported in body circulation, with reduced susceptibility to degradation (Zhou et al., 2018). miRNAs such as miR-21, miR-208, and miR-320 are varyingly associated with acute CRS (Huang et al., 2020). Interestingly, one such in miRNA abundance, miR-21, is upregulated in the stressed heart and kidney, promoting fibrosis (Kumarswamy et al., 2012; Liu et al., 2016). miR-21 is detected in the urinary compartment in cardiac surgery patients and is also evident in AHF and AKI (Du et al., 2013; Sun and Lerman, 2019). Its pro-fibrotic characteristics have been studied recently as a potential target for CRS diagnosis and therapeutics (Kumarswamy et al., 2012; Huang et al., 2020). As described previously, hypertension is one of the most common comorbidity conditions in acute CRS. miRNA-21, miRNA-93, and miRNA-200b have been found in hypertensive patients (Kwon et al., 2016) and can be evaluated for diagnosis of CRS as well.

## 7 Challenges in identifying biomarkers

The pathogenesis of CRS lies in reduced cardiac ejection, resulting in increased central venous pressure (CVP). Increased CVP leads to insufficient renal blood flow (RBF) and activates classic hemodynamic mechanisms (Ronco et al., 2012a). However, implications of non-hemodynamic pathways, such as dysfunctional sympathetic nervous system (SNS) activation, persistent renin-angiotensin-aldosterone system (RAAS) activation, and ROS/NO imbalance, exacerbating the inflammatory and immune signalling pathways, are also operative in CRS (Ronco et al., 2012a; Haase et al., 2013). Every nation, including India, uses eGFR, sCr, and BUN as the cornerstone of clinical diagnosis of AKI and CRS in patients with HF. sCr is not an early diagnostic marker for AKI in CRS patients and eGFR is calculated using creatinine levels in serum or urine (Bragadottir et al., 2013). Moreover, the means of assessing eGFR have been poor in terms of early AKI assessment (Bragadottir et al., 2013). Although conclusive studies are lacking, serum and urine NGAL has recently been found to be elevated in early AKI in patients with ADHF. However, non-hemodynamic pathways such as dysfunctional sympathetic nervous system (SNS) activation, persistent renin-angiotensin-aldosterone system (RAAS) activation, and ROS/NO imbalance exacerbating the inflammatory and immune signalling pathways are also operative in CRS (Ronco et al., 2012a; Haase et al., 2013).

## 8 Need of novel biomarkers

CRS has emerged as a major health issue considering the robust modern-day lifestyle, and is the primary cause of baseline risk factors such as hypertension, diabetes mellitus, coronary artery diseases, and atrial fibrillation (He et al., 2021). Acute HF or CKD patients admitted with such risk factors are correlated with worsened pathophysiologic conditions in CRS patients. The lack of prognostic features leads to delayed diagnosis of the syndrome, further affecting the patient, even if certain kidney injuries or cardiac injury biomarkers are nowadays used for diagnosis of CRS. Biomarkers, such as serum creatinine, used as kidney injury markers, BNP/NT-pro BNP, or cardiac Troponin T levels used for myocardial injury, have several limitations for projecting CRS progression in a chronic setting (Goffredo et al., 2021). However, in recent years, research is growing in terms of assessing biomarkers of AKI, AHF, or CRS for simple and highly predictive biomarker searching. Novel biomarkers such as hepcidin, soluble urokinase-type plasminogen activator receptor (suPAR), placental growth factor (PlGF), urinary podocin/creatinine ratio (UP/Cr), and urinary cofilin-1 have been found in CRS patients (Nakada et al., 2019; Kim, 2020; Nikorowitsch et al., 2020; Gembillo et al., 2021). However, the poor prognostic value of such traditional biomarkers demands novel biomarkers specific to CRS, not only for better prognosis but also for greater understanding of the molecular pathophysiology. Nonetheless, given the wide and overlapping pathophysiological nature of cardiorenal syndrome, high throughput multi-omics, specifically the proteomic approach, holds great potential for improving disease prognosis and management. In this context, a multi-omics–based clinical study for the discovery of CRS specific biomarkers has been recently funded by the Indian Council of Medical Research (ICMR, Government of India). With ongoing efforts for masterful control of the pathological progression of CRS, an omics-based approach is the need of the hour for finding suitable and potential prognostic and diagnostic biomarkers with better sensitivity and specificity.

## Abbreviations

ACS, acute coronary syndrome; ADHF, acute decompensated heart failure; AHF, acute heart failure; AKI, acute kidney injury; BUN, blood urea nitrogen; BNP, B-type natriuretic peptide; CO, cardiac output; CRS, Cardiorenal syndrome; CVP, central venous pressure; CysC, cystatin C; eGFR, estimated glomerular filtration rate; ECM, extracellular matrix; HF, heart failure; LV, left ventricle; MI, myocardial infarction; NGAL, neutrophil gelatinase-associated lipocalin; NT-proBNP, N-terminal-proBNP; RAAS, renin-angiotensin-aldosterone system; RBF, renal blood flow; ROS, reactive oxygen species; SNS, sympathetic nervous system; sCr, serum creatinine; WRF, worsening renal function.
